# Evaluation of the myogenic effects of subthalamic nucleus deep brain stimulation at near therapeutic amplitudes

**DOI:** 10.3389/fnins.2026.1733633

**Published:** 2026-01-29

**Authors:** Carmen Toth, Brett A. Campbell, Leonardo Favi Bocca, Kyle Baker, Olivia Hogue, Jakov Tiefenbach, Jeffrey Negrey, David Cunningham, André G. Machado, Kenneth B. Baker

**Affiliations:** 1 Department of Neurosciences, Lerner Research Institute, Cleveland Clinic, Cleveland, OH, United States; 2 Case Western Reserve University School of Medicine, Cleveland, OH, United States; 3 Center for Neurological Restoration, Neurological Institute, Cleveland Clinic, Cleveland, OH, United States; 4 Department of Neurosurgery, Neurological Institute, Cleveland Clinic, Cleveland, OH, United States; 5 Department of Physical Medicine and Rehabilitation, MetroHealth Medical Center, Cleveland, OH, United States

**Keywords:** deep brain stimulation (DBS), motor evoked potential (MEP), Parkinson ‘s disease, standard of care (SOC), subthalamic nucleus (STN)

## Abstract

**Introduction:**

Subthalamic nucleus deep brain stimulation (STN-DBS) is a standard-of-care (SoC) treatment for the motor symptoms of Parkinson’s disease (PD); however, how therapeutic DBS influences motor output is incompletely understood. Specifically, the extent of electromyographic (EMG) modulation during DBS at therapeutic (or SoC) amplitude and its relationship to activity state could be better characterized.

**Methods:**

We studied the effects of DBS on muscle activity in sixteen participants receiving STN-DBS (in 17 stimulated hemispheres) by recording EMG activity in bilateral biceps, triceps, flexor carpi radialis, and extensor digitorum communis muscles. Data was acquired in the resting state and while participants alternated between isometric contraction and brief relaxation. We recorded EMG activity during low-frequency stimulation at participant-specific therapeutic amplitude and during stimulation using pulse amplitudes slightly above (125%) and below (75%) this level. Stimulus-locked responses from each condition were evaluated for the presence of a myogenic evoked potential.

**Results:**

DBS at therapeutic amplitude elicited a myogenic response almost exclusively in the contralateral upper extremity (CUE), with at least one response occurring in 16 out of 17 hemispheres. Myogenic responses, which typically started between 10-30 milliseconds and often lasted until ~70-150 milliseconds post-stimulation, were more common in biceps/triceps. Responses were more prevalent during active contraction compared to relax/rest states as stimulation amplitude increased.

**Discussion:**

These findings support that STN-DBS-induced myogenic activity is commonplace at therapeutic stimulation amplitudes used clinically; thus, studies evaluating the degree to which myogenic effects during SoC STN DBS are associated with the clinical and side effects of STN DBS therapy are warranted.

## 1 Introduction

Subthalamic nucleus deep brain stimulation (STN DBS) is a standard of care (SoC) treatment for the motor symptoms of Parkinson’s disease (PD) (Deuschl et al., 2006). Examining how DBS affects peripheral muscle activity may help elucidate its therapeutic vs. side effect (SE) inducing neuro-modulatory mechanisms. It is well-established clinically that spread of electrical current to the internal capsule (IC) can elicit unwanted motor SEs (mSEs) appreciable as visible contraction of facial and contralateral upper extremity (CUE) musculature (Ashby et al., 1999; Mahlknecht et al., 2017; Nikolov et al., 2022). Myogenic activity can be observed on electromyography (EMG) during low frequency STN DBS as myogenic evoked potentials (MEPs), with onset times ranging from 10 to 30 msec post-stimulation and multi-phasic activity often lasting beyond 100 msec (Ashby et al., 1999; Mahlknecht et al., 2017; Nikolov et al., 2022; Testini et al., 2025). Averaging of repeated pulse deliveries allows for the appreciation of sub-clinical muscle activation (as MEPs) using amplitudes below those associated with overt, clinical mSEs (Testini et al., 2025). These findings support that even lower amplitude DBS settings, including those used clinically for therapeutic benefit, may induce myogenic effects without SEs, in contrast to higher DBS intensities.

The dependence of DBS-MEPs on stimulation amplitude and motor state has been previously demonstrated, though never during near-therapeutic amplitudes, and often with an emphasis on pulse amplitudes associated with clinical mSEs (Mahlknecht et al., 2017; Nikolov et al., 2022; Karazapryanov et al., 2024). In general, responses have been shown to increase with increasing stimulus amplitude (Ashby et al., 1999) and to be sensitive to muscle state, with greater MEP response during contraction compared to resting state (Mahlknecht et al., 2017; Compta et al., 2006). Given evidence of MEPs occurring at therapeutic amplitudes (Ashby et al., 1998; Ashby et al., 1999), detailed investigation under near-therapeutic conditions is warranted as evidence of a consistent pattern of MEP formation associated with therapeutic benefit or clinical mSEs could help guide therapeutic DBS parameter optimization.

In this study, we examined MEP prevalence in the muscles of bilateral upper extremities during DBS at therapeutic amplitudes which would be applied as SoC. To evaluate for any significant relationship between MEP formation and external parameters, we further studied the effects of slight perturbations in stimulation amplitude around the therapeutic level and of motor status (i.e., contraction versus rest) on MEP prevalence. Finally, we quantify the temporal characteristics (i.e., onset and offset latency and morphology) and somatotopic distribution (i.e., prevalence in proximal vs. distal muscles) of MEPs.

## 2 Materials and methods

All study-related procedures were performed according to a protocol approved by the Institutional Review Board of the Cleveland Clinic, under the clinical trial NCT04361955 and with additional data from individuals enrolled in NCT04563143.

### 2.1 Study participants

Individuals who were at least 5 months post-STN DBS implantation for standard of care treatment of idiopathic PD were recruited. Participants prescribed anti-parkinsonian medication could be on medication during data acquisition.

### 2.2 Electrical stimulation

DBS was delivered using the lead contralateral to the more symptomatic hemibody (if implanted bilaterally). Stimuli were delivered using each participant’s implantable pulse generator (IPG) after confirming hardware and connection integrity via impedance check. Parameters were derived from SoC settings; however, for individuals whose SoC configuration was monopolar (i.e., used the IPG as the return), we converted to a bipolar montage to minimize electrical artifact. Specifically, the cathode was preserved, with the anode set as the contact, annular or pseudo-annular, immediately dorsal to the cathode. If the SoC cathode was the most dorsal contact, the anode was instead set to the contact immediately ventral to that location. In one instance, impedance issues required moving the re-configured anode further away from the SoC cathode. In all cases, after re-configuration, pulse amplitude was increased by 25% to account for the change in current spread as reported previously (Hancu et al., 2019). Acute side-effects were ruled out for each amplitude setting by repeated, non-ramped activation of stimulation using 130 Hz stimulation and their clinically applied pulse width (which was 60 μsec in most participants, though it varied between 50 and 90 μsec across the cohort). Subsequent experiments employed 6 Hz DBS at the clinically applied pulse width, with DBS pulse amplitudes applied at therapeutic (“tx”; 100% SoC) as well as a sub- (“sub-tx”; 75% SoC) and super-therapeutic (“super-tx”; 125% SoC) pulse amplitudes. The sub- and super-therapeutic levels were included to help understand the relative proximity of the therapeutic level settings to inducing MEP activity, with the lower and higher amplitudes expected to show correspondingly lower and higher MEP prevalence, respectively.

### 2.3 Electrophysiology data acquisition

Electromyography was recorded using adhesive, hydrogel surface electrodes (Medsource Labs, Chanhassen, MN, USA) placed bilaterally over the biceps, triceps, flexor carpi radialis (FCR), and extensor digitorum communis (EDC) muscles. A ground electrode was placed on the sternum, and additional scalp electrodes were used to isolate the timing of DBS pulse delivery via electrical artifact (see Section 2.5). Data were acquired at a sampling rate of 5,000 Hz with acquisition bandpass filtering between 0.1 and 1,000 Hz (BrainAmp DC64, BrainVision LLC, NC, USA).

### 2.4 Isometric block tracking motor task

**Figure 1:**
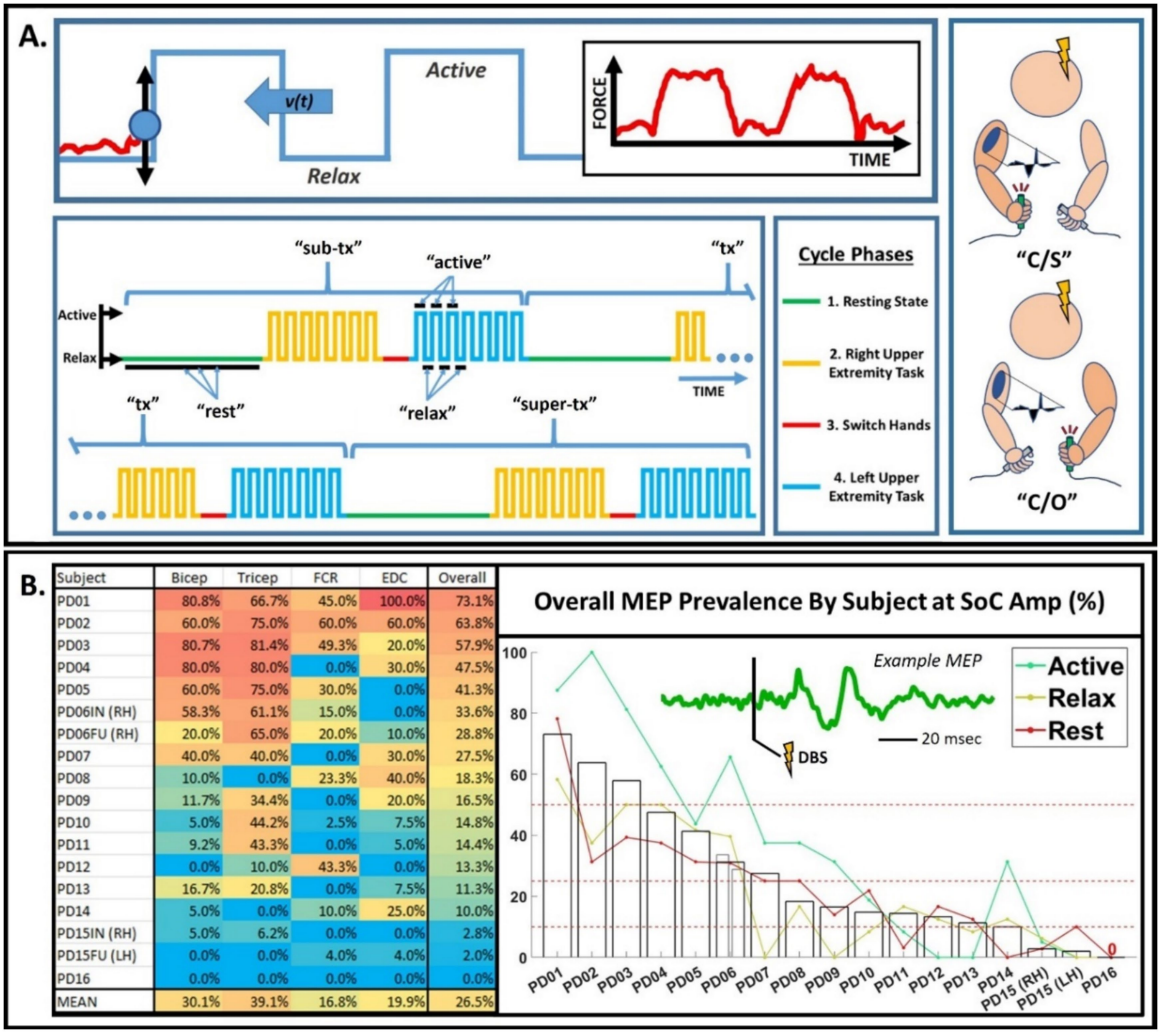
**(A)** Block tracking experiment. During the task, the patient moves a ball up and down by squeezing a dynamometer to align it with a square wave passing by from right to left (shown on the left), leading to alternating periods of contraction and relaxation (shown on the right). The task was organized into cycles of four phases: resting state, task with right hand, switch hands, and task with left hand. The above cycle was repeated, where each time DBS was administered at different stimulation amplitudes relative to the standard-of-care level. The task-related data could be divided into “Contralateral/Same” and “Contralateral/Opposite” condition groups (“C/S” and “C/O′, respectively). **(B)** MEP prevalence by participant and muscle group during DBS at SoC (therapeutic) amplitude. Table displaying percentage of trials classified as MEPs (i.e., “prevalence”) for each participant overall and as a function of muscle group, with motor state conditions lumped (left). One participant (PD06) had an initial (IN) and follow up (FU) recording session with stimulation in the same hemisphere on separate days; the rest had all data taken within one session per hemisphere. The table is color-coded such that red represents a higher prevalence and blue represents a lower prevalence. Bar graph visually depicting the distribution of MEP prevalence across participants, from the most to the least responsive participant, with an example MEP as inlet (right). Participant codes were artificially renamed in the order of the distribution in a post-hoc manner. The light gray, thin bars show the results on separate days (IN/FU) for PD06, who had multiple sessions in the same hemisphere. Solid blue-green, tan, and red lines represent the MEP prevalence seen in the Active, Relax, and Rest state for each participant, respectively. Dotted red lines transect prevalence values of 50, 25, and 10%. RH, right hemisphere; LH, left hemisphere.

Participants were seated upright and asked to perform a visually guided isometric block-tracking task. The participant applied grip force to a dynamometer (model HDBTA, Vernier) to guide a computer-generated ball along a right-to-left scrolling, 0.1–0.2 Hz square wave whose “high” and “low” phases were calibrated to 30% (“Active”) and 0% (“Relax”) of maximum voluntary contraction, respectively (Gopalakrishnan et al., 2022; see Figure 1 and Section 2.4 of the Supplementary material). Interleaved 1–2-min resting periods (“Rest”), where the dynamometer was released, occurred between each task repetition. The absence of task engagement during “Rest” thus differentiates it from task related “Relax” phase.

Task performance alternated between the two UEs to evaluate for bilateral effects of unilateral DBS, yielding two task-related groups for each recorded UE (Figure 1). The “Contralateral/Same” condition (“Active” and “Relax”) refers to recordings in the CUE while that same extremity performs the task. The “Contralateral/Opposite” conditions (“Opposite Active” and “Opposite Relax”) refer to recordings in the CUE while the task is performed with the ipsilateral upper extremity (IUE). “Rest” corresponds to neither condition; instead serving as a control for each task-related condition group. EMG recordings were also taken from the IUE and divided similarly.

### 2.5 Signal processing

EMG recordings were stratified by motor state/pulse amplitude and epoched to align with the individual stimulation events using the Fieldtrip toolbox (Oostenveld et al., 2011) (Section 2.5 of the Supplementary material). A given condition could contain a variable number of epochs, from several hundred to several thousand (median: 504, 2.5th-97.5th percentile: 148–1883). Thus, to balance signal-to-noise ratio (SNR) in each condition with sufficient SNR to permit accurate MEP detection, up to ten trials were formed per condition by averaging unique sets of 150 epochs, randomly subsampled without replacement. If less than 150 epochs existed (which occurred in ~3% of conditions), one trial was formed by averaging all epochs.

#### 2.5.1 Classification and extraction of DBS-MEPs

Trials were reviewed and classified according to the presence or absence of a DBS-MEP. We employed a modified z-scoring metric ($ZS_{\mathrm{MOD}}$) previously applied in the literature (Ziemann et al., 1999), defined as:

$$ZS_{\mathrm{MOD}}=\frac{\mathrm{MAX}W_{\text{RESPONSE}}-\mu_{\text{BASELINE}}}{\sigma_{\text{BASELINE}}}$$

Where $\mathrm{MAX}W_{\text{RESPONSE}}$ is the maximum amplitude the rectified trial waveform in the response period (+10 to +120 msec post-stimulation) exceeded over a one millisecond window and $\mu_{\text{BASELINE}}$ and $\sigma_{\text{BASELINE}}$ are the mean and standard deviation of the rectified baseline period (−40 to −3 msec pre-stimulation), respectively. Trials where $ZS_{\mathrm{MOD}}\geq 4$ were initially classified as MEPs. A visual review was conducted to refine the results. Specifically, trials initially classified as MEPs were reclassified as null if, for example, if there was a large noise spike in the response period. Trials initially classified as null could be reclassified as MEPs if there was a large noise spike in the baseline period or if subtle modulatory activity consistent with other trials/conditions was identified.

### 2.6 Determination of MEP prevalence across participants at therapeutic amplitude

We defined “MEP prevalence” as the percentage of trials classified as MEPs and quantified that value for each participant at the therapeutic (“tx,” or SoC) amplitude. MEP prevalence within a participant was determined by first finding the MEP prevalence within each muscle and motor state, then averaging these within-condition values across conditions to weigh each equally. This weighted average specifically controls for the varying number of trials in different conditions.

### 2.7 Determination of temporal characteristics of MEP responses across muscles

We analyzed MEP onset and offset latency (or the times when MEP activity began and ceased, respectively) by averaging all MEP trials within a condition, then manually labelling the onset and offset times of the condition-wide average MEP. Only the therapeutic amplitude was considered for this analysis, but all motor states and muscles were evaluated in all participants.

### 2.8 Statistical analyses

Differences in CUE MEP prevalence between conditions were statistically evaluated by analyzing individual trials as binary observations (“MEP/1” or “Null/0”). MEP prevalence was compared between motor states and stimulation amplitudes using generalized linear mixed effects models (GLMM) with logit links. GLMM are flexible extensions of linear regression that can accommodate repeated measurements per participant, unbalanced data, and non-Gaussian response data. Each model included a random intercept for participant and a compound symmetry covariance matrix was implemented. Results from each model are expressed as odds ratios with 95% confidence intervals.

The same analyses were also used to compare MEP prevalence within distal (FCR/EDC) versus proximal (biceps/triceps) muscles with all motor states combined and in each motor state, with amplitudes lumped.

The above analyses were carried out using SAS Studio v3.7 and were two-tailed (*α* = 0.05). All analyses were completed separately for the Contralateral/Same and Contralateral/Opposite datasets.

Given the exploratory nature of the study, correction for multiple comparisons was not applied.

## 3 Results

The main findings reported here are that: (1) MEPs were common in our cohort (16/17 hemispheres) during DBS at near-clinical, non-mSE inducing parameters, (2) MEPs were found only in the CUE and predominantly in proximal muscles, and (3) MEP prevalence was dependent on motor state (Active > Rest) in the near-therapeutic (or SoC) amplitude range.

### 3.1 Patient demographics/characteristics

Data were collected from 16 participants (seven female, mean age: 64.2 years, mean disease duration: 9.6 years). PD06 and PD15 returned for a follow-up visit to acquire missing data for the supra-therapeutic amplitude condition. Because data were collected from each of PD15’s bilateral implants in subsequent sessions, those were classified as independent samples, yielding 17 total hemispheres. Additional demographic details are provided in Table 1.

**Table 1:** Patient characteristics.

Subject*	Age (years)	Disease duration (years)	Sex	Implant Side	UPDRS III Score (OFF DBS/OFF MEDS)			Pre-Op Approximate LEDD (mg)
					Pre-Op	Post-Op	%∆	
PD01	53	21	M	Left	32	30.5	−1.5 (5%)	1,403
PD02	72	15	F	Right	30	16	−14 (47%)	1,040
PD03	72	5	F	Left	39	25	−14 (36%)	600
PD04	48	7	M	Right	41	30	−11 (27%)	1,336
PD05	67	13	F	Left	33	28	−5 (15%)	942
PD06^a^	61/62	12/13	F	Right/Right	40	37.5	−2.5 (6%)	978
PD07	69	13	F	Left	25	14	−11 (44%)	1,030
PD08	69	5	M	Left	42	43	+1 (2%)	600
PD09	57	3	F	Right	31	24.5	−6.5 (21%)	750
PD10	68	5	M	Right	23	9	−14 (60%)	600
PD11	54	10	M	Left	44	33.5	−10.5 (24%)	1,472
PD12	65	7	M	Left	52	45	−7 (13%)	1,010
PD13	74	7	M	Right	27	21	−6 (22%)	1,114
PD14	62	13	M	Right	40	20	−20 (50%)	900
PD15^a^	74/75	13/14	M	Right/Left	37.5	27	−10.5 (28%)	870
PD16	63	5	F	Left	33	46	+13 (39%)	1,043

Subject*	SoC stimulation amplitude (mA)	MEP prevalence at SoC amplitude (%)	Lead Type	Lead manufacturer	MNI^b^ Coordinates of Cathode (mm)		
					X	Y	Z
PD01	4.40	73.1	6,172	St Jude	−13.6	−13.2	−6.9
PD02	4.50	63.8	6,173	St Jude	14.0	−14.4	−6.3
PD03	2.95	57.9	6,172	St Jude	−12.9	−14.2	−5.3
PD04	3.00	47.5	Vercise Cart.	Boston Sci.	10.9	−12.2	−6.9
PD05	2.75	41.3	Vercise Cart.	Boston Sci.	−11.1	−15.9	−9.1
PD06^a^	4.30/4.30	33.6/28.8	Vercise	Boston Sci.	14.8	−10.3	−3.9
PD07	1.75	27.5	B33005	Medtronic	−11.8	−14.3	−6.9
PD08	1.55	18.3	6,172	St Jude	−12.3	−15.5	−7.7
PD09	3.50	16.5	6,172	St Jude	12.9	−14.0	−4.8
PD10	2.70	14.8	6,172	St Jude	12.8	−12.8	−8.0
PD11	4.40	14.4	6,172	St Jude	−12.2	−11.9	−5.2
PD12	1.00	13.3	B33005	Medtronic	−12.9	−15.3	−6.7
PD13	1.75	11.3	6,172	St Jude	16.3	−11.1	−2.3
PD14	3.90	10.0	6,172	St Jude	13.1	−11.7	−5.0
PD15^a^	3.85/4.25	2.8/2.0	3,389	Medtronic	14.6/−13.6	−12.5/−10.3	−5.6/−5.7
PD16	2.50	0.0	B33005	Medtronic	−12.5	−12.9	−6.4

*Subjects were renamed from highest to lowest based upon overall MEP prevalence as PD01-PD16. Thus, the variables in this table can be readily correlated (approximately) with overall MEP prevalence at SoC (or therapeutic) amplitude accordingly.

a For subjects PD06 and PD15, where results are listed as “X/Y,” these values correspond to the initial and follow up sessions, respectively. PD15’s sessions are from separate hemispheres; PD06’s are not.

b MNI 152 Nonlinear 2009b Asymmetric space as per reconstruction in Lead-DBS (Horn et al., 2017). Note that dorsolateral STN is located at approximately (x, y, z) = (±11 to ±14, −12 to −15, −5 to −9) mm.

Data was acquired at all three amplitudes in only five participants; a larger, overlapping cohort of fourteen participants had data at sub-tx and tx amplitudes. Statistical comparisons involving super-tx data were thus evaluated in only five participants, while all other comparisons included the larger cohort (Section 2.8).

### 3.2 Distribution of MEPs across participants at therapeutic amplitude

A consistent pattern of MEP formation during DBS at therapeutic (SoC) amplitude was only detected in the CUE. Figure 1 summarizes CUE MEP prevalence during therapeutic-amplitude DBS by participant, including both overall and muscle group-specific values. Considering all muscles, at least one CUE MEP response was observed for every study participant except one (PD16), however prevalence values varied widely across participants (0–73.1%; overall mean: 26.5%). All responding participants showed a response in at least two muscles. The highest average prevalence was observed in the triceps (39.1%), followed by biceps (30.1%), EDC (19.9%), and finally the FCR (16.8%).

### 3.3 Distribution of MEPs across proximal and distal upper-extremity musculature

**Figure 2:**
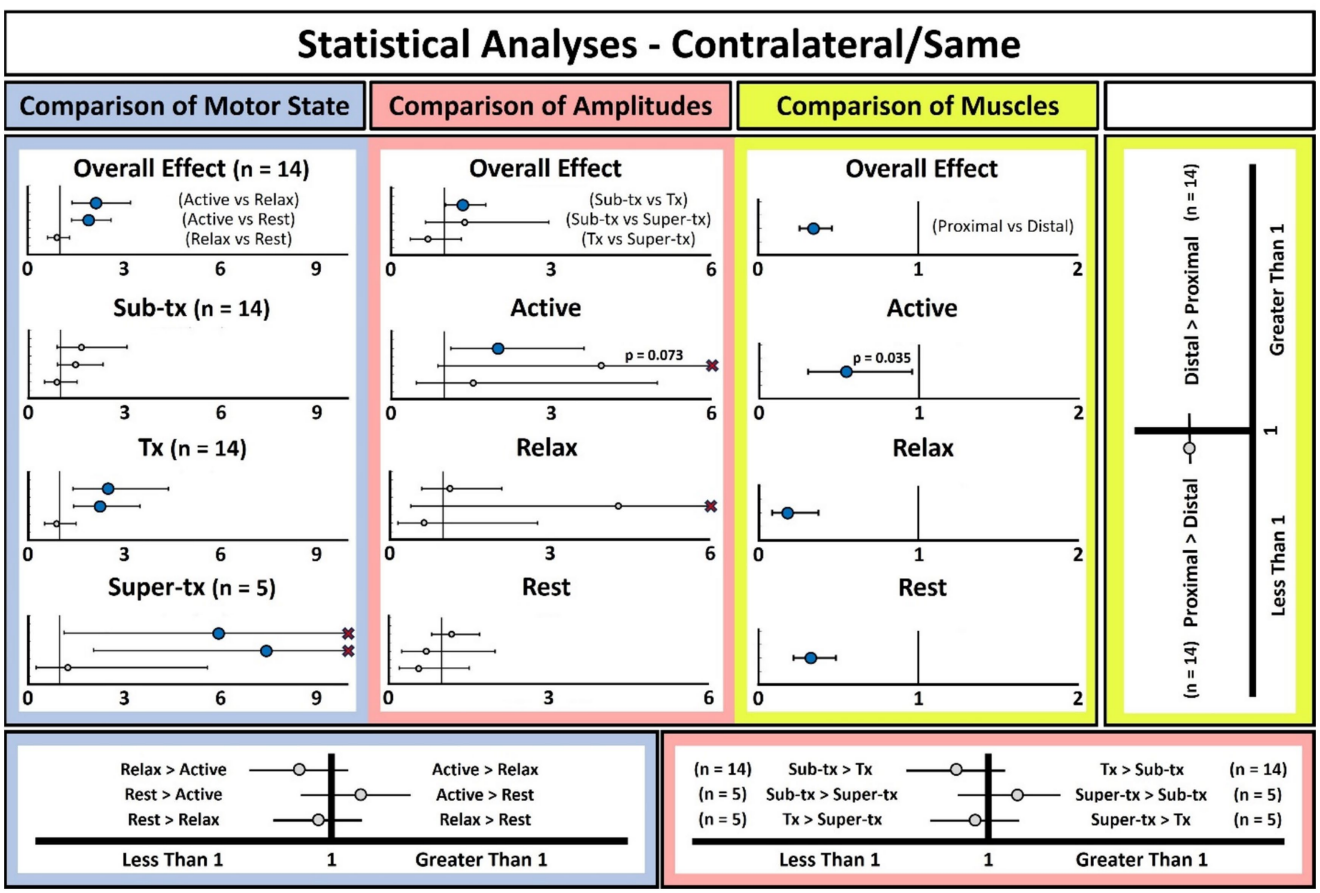
Effect of motor state, stimulation amplitude, and somatotopy (i.e., muscle) on MEP prevalence in contralateral/same condition. Statistical analysis of the effect of motor state (i.e., active vs. relax vs. rest) at different stimulation amplitudes is shown in the blue panel (upper row, first from left) as odds ratios, and the results are further explained by the blue key (bottom row, left). Note that the results in the blue “Overall Effect” panel are drawn from the larger pool of participants but only includes data at the sub-Tx and Tx amplitudes; however, the analysis in five participants including all amplitudes yielded concordant results (see Supplementary Table S1). Statistical analysis of the effect of stimulation amplitudes (sub-Tx vs. Tx vs. super-Tx) in different motor states is shown in the red panels (upper row, second from left) as odds ratios and corresponds to the red key (bottom row, right). Statistics to determine whether MEP prevalence differed between proximal (i.e., biceps and triceps) and distal (i.e., FCR and EDC) muscles is shown in the yellow panels and legend (upper row, right). Results were performed in the larger group of participants (*n* = 14) and so only incorporate data at the sub-Tx and Tx amplitudes. Large blue circles represent significant comparisons at *α* = 0.05, whereas small white circles are non-significant, and horizontal bands represent 95% confidence intervals. Note that all statistical comparisons involving data from the super-Tx condition have less statistical power, as there were only five participants with such data. As such, the confidence intervals were wide and did not always fit inside the plotted range (marked by thin red X’s). To see the full confidence intervals, please refer to Supplementary Table S2.

MEP prevalence was significantly higher in proximal relative to distal musculature regardless of motor state and for both Contralateral/Same (Figure 2; Active: *p* = 0.035, Relax: *p* < 0.0001, Rest: *p* < 0.0001) and Contralateral/Opposite (Supplementary Figure S1; Active: *p* = 0.0001, Relax: *p* = 0.0006, Rest: *p* < 0.0001) conditions. Within the Contralateral/Same condition, the difference detected in the Active state remained significant, however the magnitude of the difference was reduced relative to the Relax/Rest states. This effect was not observed in the Contralateral/Opposite condition group.

### 3.4 Effect of muscle contraction and stimulation amplitude on MEP formation

When performing the task with the CUE and considering the Contralateral/Same condition group, MEP prevalence was increased significantly in Active relative to both the Relax and Rest states. This was the case both when the data were collapsed across all pulse amplitude conditions (Active vs. Relax: *p* = 0.0005 and Active vs. Rest: *p* = 0.0001) and, specifically, within the tx (Active vs. Relax: *p* = 0.002 and Active vs. Rest: *p* = 0.0004) and super-tx (Active vs. Relax: *p* = 0.034 and Active vs. Rest: *p* = 0.003) amplitude conditions (Figure 2; Supplementary Tables S1, S2). MEP prevalence also was increased for the tx amplitude relative to sub-tx when collapsed across motor states (*p* = 0.033) and only sustained in the sub-analysis comparing the two pulse amplitude conditions with the Active state (*p* = 0.003). No pulse amplitude-related differences were observed within the Relax and Rest states.

Results in the Contralateral/Opposite condition group are presented in Supplementary Figure S1 and Section 3.3 of the Supplementary material.

### 3.5 Temporal distribution of MEP waveforms across muscles

**Figure 3:**
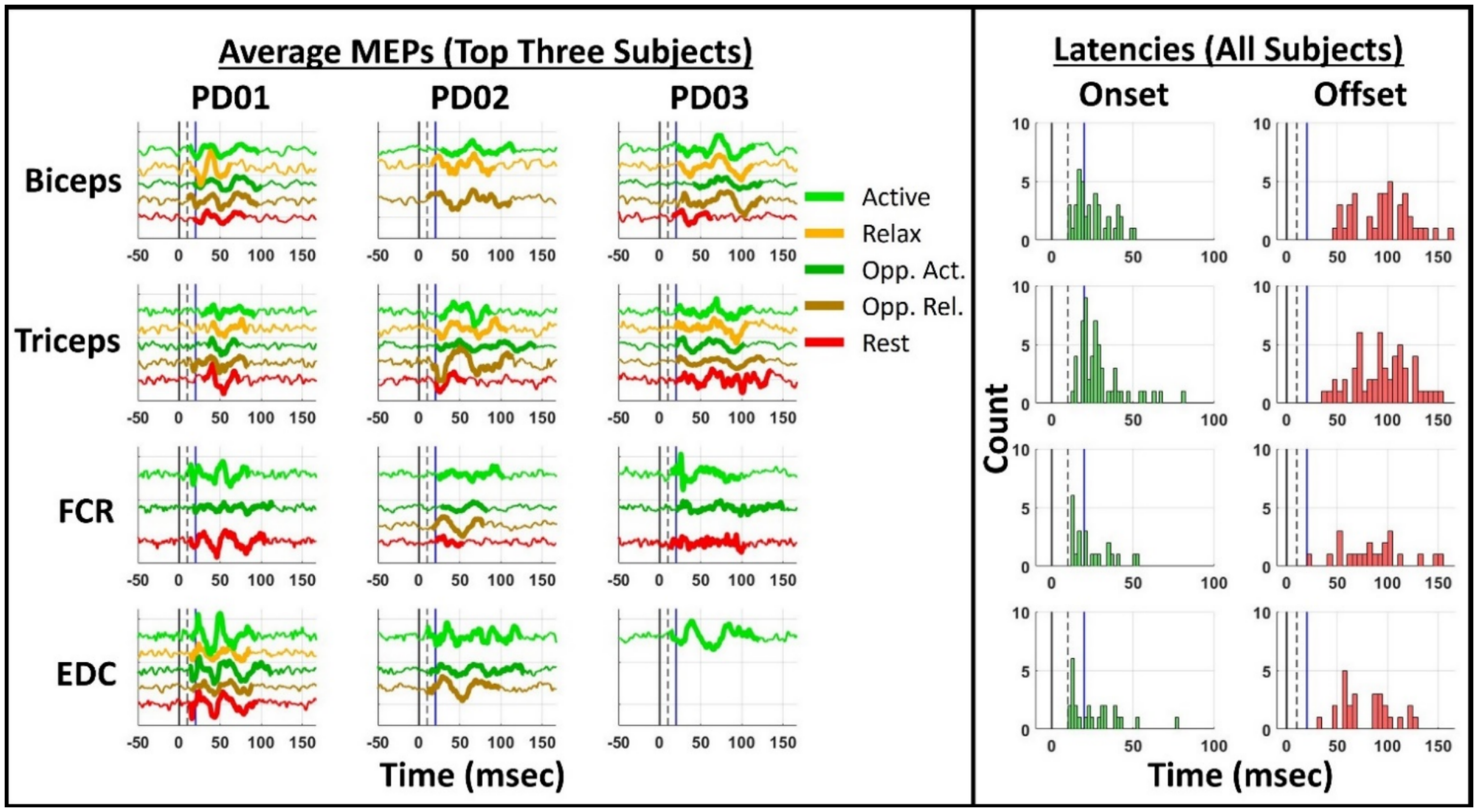
Representative MEP waveform morphologies and onset/offset latencies at SoC stimulation amplitude. Average MEP responses are plotted from −50 to +167 ms in all motor states in the three most responsive participants (PD01–03) for each muscle (left). Active state MEPs are colored light green (top waveform), Relax state MEPs are colored orange (upper-middle waveform), Opposite Active MEPs are colored dark green (middle waveform), Opposite Relax MEPs are colored brown (lower-middle waveform), and Rest state MEPs are colored red (bottom waveform). The time period containing MEP activity is highlighted (thicker line). If a MEP waveform is not plotted, this means that no MEPs were found in that participant/condition. Histograms of MEP onset (green, left column) and MEP offset (red, right column) latencies are plotted, where onset is the “MEP start time” and offset is the “MEP stop time” (right). For both panels, the time/latency of +0 ms is marked as a solid black vertical line, +10 ms is plotted as a dotted black vertical line, and +20 ms is marked as a solid blue line.

CUE MEPs showed a consistent response pattern in all muscles, manifesting between 10 and 160 msec after stimulation typically, but not exclusively, as a triphasic-patterned response. MEPs generally exhibited similar morphology regardless of motor state. MEPs often had an onset of 10–30 msec, with offset times varying widely between 50 and 150 msec. Prolonged onset times (>30 msec) and durations (>100 msec) were also common, especially in proximal muscles (Figure 3). Representative MEPs in all sixteen participants are shown in Supplementary Figure S2.

## 4 Discussion

### 4.1 Subclinical myogenic activation may be commonplace in clinical DBS

This study identified MEPs in CUE muscles at therapeutic amplitude in nearly all participants (Figure 1), which suggests that subtle EMG modulation may be commonplace in clinical DBS. These findings are consistent with Ashby et al. (1998) and Ashby et al. (1999), who demonstrated that GPi DBS at therapeutic amplitudes could evoke myogenic activity in one participant and that therapeutic-amplitude STN DBS produced CUE MEPs in 7 out of 23 hemispheres across 12 patients. Our findings suggest a higher prevalence of MEPs at near-therapeutic amplitudes, which could be explained by the higher signal-to-noise ratio afforded by our larger epoch count (150 versus 50–100 epochs). The common occurrence of MEPs at therapeutic amplitude is also consistent with recent data showing that STN DBS MEPs could be elicited in the absence of SEs in 11 of 12 participants (Testini et al., 2025). Our work builds upon those prior observations, characterizing the prevalence of myogenic activity at pulse amplitudes consistent with therapeutic stimulation levels in a relatively large cohort while also revealing the high degree of response variability (0–73.1%) across individuals.

### 4.2 MEPs were exclusively contralateral and proximal muscle dominant

We found that MEPs manifested consistently in the CUE (Figure 1), with no evidence of IUE MEPs at any pulse amplitude tested. This is consistent with the decussating corticospinal tract (CST) and with studies involving subcortical stimulation near the IC (Kühn et al., 2004; Compta et al., 2006; Costa et al., 2007), including Costa et al. (2007) who observed MEPs in ipsilateral facial muscles but not IUE muscles. Kühn et al. (2004) performed both transcranial magnetic stimulation (TMS) and subcortical stimulation and found IUE MEPs only during cortical stimulation, attributing the difference to activation of transcallosal pathways by cortical stimulation. Although Compta et al. (2006) found no evidence of IUE MEPs associated with STN DBS, the authors reported long-latency inhibitory silent periods in the ipsilateral thenar muscle, which suggests involvement of an indirect, polysynaptic pathway.

We found greater CUE MEP facilitation in proximal as compared to distal muscles, although MEP prevalence in distal muscles was more comparable (though still less) during the Active state specifically (possibly relating to the activity-related enhancement of MEPs during the isometric grip task preferentially recruiting distal UE muscles). Our finding of predominant MEP facilitation in proximal muscles contrasts with the greater CST representation of distal muscles (Phillips and Porter, 1964) and TMS-based MEP studies showing higher prevalence in distal musculature (Palmer and Ashby, 1992). Within DBS studies, results have been mixed (Testini et al., 2025; Costa et al., 2007; Shils et al., 2021). Consistent with our findings, Testini et al. (2025) reported a higher prevalence in biceps (40%) versus FCR (9%) during DBS at SE inducing levels. However, Costa et al. (2007) and Shils et al. (2021) found either no difference or a higher prevalence of distal versus proximal muscle activation, respectively. Differences in methodology, including the use of supra-threshold amplitudes associated with overt mSEs, burst stimulation, and study of single-event recordings may account for these disparities.

The preponderance of MEPs in proximal muscles detected here and in Testini’s study (who likewise averaged 50–150 epochs) could be due to subtle, polysynaptic modulation which cannot be detected using single-event recordings. This polysynaptic modulation could have a distinct somatotopic distribution relative to distal-predominant MEP activity resulting from TMS activation (Palmer and Ashby, 1992). This would be consistent with the prolonged onset times of our biceps/triceps MEPs and the ipsilateral silent periods detected by Compta et al. (2006). Another possibility is that CST fibers associated with proximal muscles pass closer to the STN and are thus more prone to activation from STN DBS current spread; however, existing tractography studies lack sufficient granularity to definitively address this (Petersen and McIntyre, 2023) and observations during intraoperative mapping and clinical programming do not fully support it.

### 4.3 MEPs were facilitated by active contraction in an amplitude-dependent manner

Active muscle contraction (Mahlknecht et al., 2017; Compta et al., 2006; Ashby et al., 1998) and increasing stimulation amplitude (Ashby et al., 1999; Costa et al., 2007) can facilitate MEPs. Our study revealed that active contraction increases MEP facilitation relative to the resting state, and that this difference is exacerbated further by increasing stimulation amplitudes beyond the SoC level. We also found no difference in MEP prevalence between short periods of rest which are interleaved with periods of motor engagement (Relax) and extended resting state periods (Rest). Recent studies propose that MEPs may be valuable in DBS clinical practice to promote real-time feedback regarding IC proximity and side effect risk, thereby enabling device repositioning (Nikolov et al., 2022; Karazapryanov et al., 2024; Testini et al., 2025). Interestingly, Testini and colleagues found that MEP prevalence was more relevant than MEP amplitude in determining the relationship between MEPs and SEs (Testini et al., 2025).

The above suggests that MEPs could hold clinical value during DBS optimization. Specifically, by quantifying MEP prevalence in resting versus active states across a set of test amplitudes, either emerging activity-related changes and/or absolute changes in either motor state could be used to predict the side effect-inducing threshold. Although more data are needed, it is possible that MEPs could be a useful biomarker in helping to predict an ideal therapeutic amplitude (i.e., one that alleviates symptoms while being below SE threshold) to help further refine the DBS therapeutic window. Such a method could be incorporated into a broader machine learning-based algorithm which also leverages external parameters, such as device location, other stimulation parameters, and patient clinical status. Although our study does not explore the presence or absence of a relationship between MEP prevalence during therapeutic amplitude DBS and clinical benefit, the existence of a relationship would further strengthen the utility of MEPs during clinical programming. Several past studies support a relationship between improvement in rigidity and IC fiber activation, of which MEPs are a known effect (Xu et al., 2011; Butson et al., 2011; Johnson et al., 2012). However, the effectiveness of MEPs in clinical programming is not substantiated directly by our findings and may in fact only be valuable for a sub-population of patients with PD (such as those with higher MEP prevalence) if any. If this sub-population could be predicted in advance using external parameters including those described above; however, there may still be practical value in developing this method.

### 4.4 MEP onset and duration: possible evidence for activation of multiple pathways

We detected extended (>70–100 msec), multi-phasic MEPs and report onset times (10–30 msec) consistent with prior studies (Ashby et al., 1999; Compta et al., 2006; Costa et al., 2007; Mahlknecht et al., 2017; Nikolov et al., 2022; Testini et al., 2025). These earlier onset times, despite unexpectedly wide variance (likely due to the subtle nature of the responses elicited by sub-SE threshold DBS), are consistent with direct activation of CST fibers by DBS current (Ashby et al., 1998, 1999; Butson et al., 2007; Tommasi et al., 2008; Mahlknecht et al., 2017; Nikolov et al., 2022). This is supported by single motor unit studies during GPi DBS (Ashby et al., 1998; Kühn et al., 2004) and STN DBS (Ashby et al., 1999), which revealed increased firing probability in the first dorsal interosseus muscle starting around 18–20 msec and lasting for only 2 msec, consistent with a monosynaptic, IC-mediated pathway. Indeed, subsequent work has shown an inverse relationship between IC activation and thresholds of MEP formation (Nikolov et al., 2022; Butson et al., 2007; Mahlknecht et al., 2017) and mSEs (Tommasi et al., 2008).

Though direct IC activation is the most probable cause of the early facilitative component of the MEP, our findings of occasionally delayed onset times (>30 msec), extended offset times (>70 msec), and multi-component MEPs suggest that the underlying mechanisms may be multifactorial and involve additional indirect, polysynaptic pathways. Ashby’s group detected MEP activity beyond 100 msec and suggested that a trough observed at 40 msec could arise from thalamocortical inhibition (Ashby et al., 1999), as they observed concurrent motor cortex (M1) activation. M1 activation could be implicated in forming the later MEP components via other mechanisms, as activation of IC can antidromically activate M1 (Costa et al., 2007; Johnson et al., 2009) and STN DBS may modulate M1 antidromically through the hyperdirect pathway (Hanajima et al., 2004). The long-latency components of the MEP could alternatively (or additionally) be caused by a rebounding spinal reflex after the initial myogenic impulse which induces a subsequent facilitative phase, as the long-latency stretch reflex is exaggerated in PD (Lee and Tatton, 1975; Rothwell et al., 1983).

## 5 Study limitations

There are several limitations to this study, which in particular may affect the scope and translational value of its findings. The conversion to a lead-bipolar configuration in individuals whose clinical settings were monopolar may limit the translatability of these findings, although we did attempt to correct for this by adjusting the pulse amplitude as described by Hancu et al. (2019). However, considering that monopolar stimulation at identical amplitude would be expected to induce more extensive current spread to IC (Chaturvedi et al., 2013), and thus more MEP activity, it would be unexpected for substantially less MEP activity to be observed under monopolar conditions. Also, the use of low frequency stimulation (~6 Hz) in lieu of the high frequencies (~130 Hz) used clinically could be a limitation, although prior studies suggest that varying frequency should not strongly affect MEP formation as varying frequency does not affect the spatial spread of stimulation (Testini et al., 2025; McIntyre et al., 2004). Another limitation of this study is that we only considered EMG data from upper extremity muscles and did not consider data from other muscles (i.e., facial, axial, and lower extremity muscles) or the central nervous system. Thus, future studies which evaluate MEPs from a broader set of muscles alongside data relating to device position, local tissue activation (i.e., VTAs), and CNS electrophysiology, would complement this one and could help to better elucidate underlying mechanisms. Although this study shows that MEPs are present during therapeutic amplitude DBS in a relatively large cohort of patients, we do not explore whether there is any relationship between the extent of MEP elicitation and either the degree of clinical benefit or the propensity for side effects to emerge at higher amplitudes. We believe there is value in further studies evaluating for the presence or absence of such relationships. Last, the fact that only five subjects contributed data at the supra-therapeutic amplitude limits the statistical power of comparisons involving this amplitude. Future studies should further evaluate how varying stimulation amplitude around the therapeutic level affects MEP activity, while both studying a wider range of amplitudes and ensuring robust statistical power at all amplitudes. This could hold value in advancing understanding of the utility of MEP activity as a biomarker to estimate the therapeutic amplitude during device programming.

## Data Availability

The raw data supporting the conclusions of this article will be made available by the authors, without undue reservation.

## References

[ref1] Ashby P. Kim Y. J. Kumar R. Lang A. E. Lozano A. M. (1999). Neurophysiological effects of stimulation through electrodes in the human subthalamic nucleus. Brain 122, 1919–1931. doi: 10.1093/brain/122.10.1919 10506093

[ref2] Ashby P. Strafella A. Dostrovsky J. O. Lozano A. Lang A. E. (1998). Immediate motor effects of stimulation through electrodes implanted in the human globus pallidus. Stereotact. Funct. Neurosurg. 70, 1–18. doi: 10.1159/000029593, 9691237

[ref3] Butson C. R. Cooper S. E. Henderson J. M. McIntyre C. C. (2007). Patient-specific analysis of the volume of tissue activated during deep brain stimulation. NeuroImage 34, 661–670. doi: 10.1016/j.neuroimage.2006.09.034, 17113789 PMC1794656

[ref4] Butson C. R. Cooper S. E. Henderson J. M. Wolgamuth B. McIntyre C. C. (2011). Probabilistic analysis of activation volumes generated during deep brain stimulation. NeuroImage 54, 2096–2104. doi: 10.1016/j.neuroimage.2010.10.059, 20974269 PMC3008334

[ref5] Chaturvedi A. Luján J. L. McIntyre C. C. (2013). Artificial neural network based characterization of the volume of tissue activated during deep brain stimulation. J. Neural Eng. 10:056023. doi: 10.1088/1741-2560/10/5/056023, 24060691 PMC4115460

[ref6] Compta Y. Valls-Solé J. Valldeoriola F. Kumru H. Rumià J. (2006). The silent period of the thenar muscles to contralateral and ipsilateral deep brain stimulation. Clin. Neurophysiol. 117, 2512–2520. doi: 10.1016/j.clinph.2006.08.005, 17008124

[ref7] Costa J. Valls-Solé J. Valldeoriola F. Rumià J. Tolosa E. (2007). Motor responses of muscles supplied by cranial nerves to subthalamic nucleus deep brain stimuli. Brain 130, 245–255. doi: 10.1093/brain/awl336, 17151002

[ref8] Deuschl G. Schade-Brittinger C. Krack P. Volkmann J. Schäfer H. Bötzel K. . (2006). A randomized trial of deep-brain stimulation for Parkinson's disease. N. Engl. J. Med. 355, 896–908. doi: 10.1056/NEJMoa060281, 16943402

[ref9] Gopalakrishnan R. Cunningham D. A. Hogue O. Schroedel M. Campbell B. A. Plow E. B. . (2022). Cortico-cerebellar connectivity underlying motor control in chronic post-stroke individuals. J. Neurosci. 42, 5186–5197. doi: 10.1523/JNEUROSCI.2443-21.2022, 35610051 PMC9236286

[ref10] Hanajima R. Ashby P. Lozano A. M. Lang A. E. Chen R. (2004). Single pulse stimulation of the human subthalamic nucleus facilitates the motor cortex at short intervals. J. Neurophysiol. 92, 1937–1943. doi: 10.1152/jn.00239.2004, 15152016

[ref11] Hancu I. Boutet A. Fiveland E. Ranjan M. Prusik J. Dimarzio M. . (2019). On the (non-)equivalency of monopolar and bipolar settings for deep brain stimulation fMRI studies of Parkinson's disease patients. J. Magn. Reson. Imaging 49, 1736–1749. doi: 10.1002/jmri.26321 30552842

[ref12] Horn A. Neumann W. J. Degen K. Schneider G. H. Kühn A. A. (2017). Toward an electrophysiological "sweet spot" for deep brain stimulation in the subthalamic nucleus. Hum. Brain Mapp. 38, 3377–3390. doi: 10.1002/hbm.23594, 28390148 PMC6867148

[ref13] Johnson M. D. Vitek J. L. McIntyre C. C. (2009). Pallidal stimulation that improves parkinsonian motor symptoms also modulates neuronal firing patterns in primary motor cortex in the MPTP-treated monkey. Exp. Neurol. 219, 359–362. doi: 10.1016/j.expneurol.2009.04.022, 19409895 PMC2730829

[ref14] Johnson M. D. Zhang J. Ghosh D. McIntyre C. C. Vitek J. L. (2012). Neural targets for relieving parkinsonian rigidity and bradykinesia with pallidal deep brain stimulation. J. Neurophysiol. 108, 567–577. doi: 10.1152/jn.00039.2012, 22514292 PMC3404794

[ref15] Karazapryanov P. A. Gabrovski K. R. Milenova Y. Pavlov V. K. Karameshev A. Damianova M. . (2024). Mapping of capsular side effects by using intraoperative motor-evoked potentials during asleep deep brain stimulation surgery of the subthalamic nucleus for Parkinson's disease. Stereotact. Funct. Neurosurg. 102, 248–256. doi: 10.1159/000539433, 38934180

[ref16] Kühn A. A. Brandt S. A. Kupsch A. Trottenberg T. Brocke J. Irlbacher K. . (2004). Comparison of motor effects following subcortical electrical stimulation through electrodes in the globus pallidus internus and cortical transcranial magnetic stimulation. Exp. Brain Res. 155, 48–55. doi: 10.1007/s00221-003-1707-y, 15064884

[ref17] Lee R. G. Tatton W. G. (1975). Motor responses to sudden limb displacements in primates with specific CNS lesions and in human patients with motor system disorders. Can. J. Neurol. Sci. 2, 285–293. doi: 10.1017/s0317167100020382, 809129

[ref18] Mahlknecht P. Akram H. Georgiev D. Tripoliti E. Candelario J. Zacharia A. . (2017). Pyramidal tract activation due to subthalamic deep brain stimulation in Parkinson's disease. Mov. Disord. 32, 1174–1182. doi: 10.1002/mds.27042, 28590508

[ref19] McIntyre C. C. Mori S. Sherman D. L. Thakor N. V. Vitek J. L. (2004). Electric field and stimulating influence generated by deep brain stimulation of the subthalamic nucleus. Clin. Neurophysiol. 115, 589–595. doi: 10.1016/j.clinph.2003.10.033, 15036055

[ref20] Nikolov P. Heil V. Hartmann C. J. Ivanov N. Slotty P. J. Vesper J. . (2022). Motor evoked potentials improve targeting in deep brain stimulation surgery. Neuromodulation 25, 888–894. doi: 10.1111/ner.13386, 33779014

[ref21] Oostenveld R. Fries P. Maris E. Schoffelen J. M. (2011). FieldTrip: open source software for advanced analysis of MEG, EEG, and invasive electrophysiological data. Comput. Intell. Neurosci. 2011:156869. doi: 10.1155/2011/156869, 21253357 PMC3021840

[ref22] Palmer E. Ashby P. (1992). Corticospinal projections to upper limb motoneurones in humans. J. Physiol. 448, 397–412. doi: 10.1113/jphysiol.1992.sp019048, 1593472 PMC1176206

[ref23] Petersen M. V. McIntyre C. C. (2023). Comparison of anatomical pathway models with tractography estimates of the pallidothalamic, cerebellothalamic, and corticospinal tracts. Brain Connect. 13, 237–246. doi: 10.1089/brain.2022.0068, 36772800 PMC10178936

[ref24] Phillips C. G. Porter R. (1964). The pyramidal projection to motoneurones of some muscle groups of the baboon's forelimb. Prog. Brain Res. 12, 222–245. doi: 10.1016/s0079-6123(08)60625-1 14202441

[ref25] Rothwell J. C. Obeso J. A. Traub M. M. Marsden C. D. (1983). The behaviour of the long-latency stretch reflex in patients with Parkinson's disease. J. Neurol. Neurosurg. Psychiatry 46, 35–44. doi: 10.1136/jnnp.46.1.35, 6842198 PMC1027261

[ref26] Shils J. Kochanski R. B. Borghei A. Candocia A. Pal G. D. Afshari M. . (2021). Motor evoked potential recordings during segmented deep brain stimulation-a feasibility study. Oper. Neurosurg. 20, 419–425. doi: 10.1093/ons/opaa414, 33428767

[ref27] Testini P. Wang A. Cole E. R. Miocinovic S. (2025). Motor evoked potentials as a side effect biomarker for deep brain stimulation. Clin. Neurophysiol. 183:2111499. doi: 10.1016/j.clinph.2025.2111499, 41455237 PMC12910348

[ref28] Tommasi G. Krack P. Fraix V. Le Bas J. F. Chabardes S. Benabid A. L. . (2008). Pyramidal tract side effects induced by deep brain stimulation of the subthalamic nucleus. J. Neurol. Neurosurg. Psychiatry 79, 813–819. doi: 10.1136/jnnp.2007.117507, 17928327

[ref29] Xu W. Miocinovic S. Zhang J. Baker K. B. McIntyre C. C. Vitek J. L. (2011). Dissociation of motor symptoms during deep brain stimulation of the subthalamic nucleus in the region of the internal capsule. Exp. Neurol. 228, 294–297. doi: 10.1016/j.expneurol.2010.08.007, 20713049 PMC3536485

[ref30] Ziemann U. Ishii K. Borgheresi A. Yaseen Z. Battaglia F. Hallett M. . (1999). Dissociation of the pathways mediating ipsilateral and contralateral motor-evoked potentials in human hand and arm muscles. J. Physiol. 518, 895–906. doi: 10.1111/j.1469-7793.1999.0895p.x, 10420023 PMC2269467

