# Supplementary material for: Evaluation of the myogenic effects of subthalamic nucleus deep brain stimulation at near therapeutic amplitudes
Source: Front Neurosci. 2026 Jan 29;20:1733633. doi: 10.3389/fnins.2026.1733633 (PMC12910608; doi:10.3389/fnins.2026.1733633)
Supplement: Supplementary file 1 [file Data_Sheet_1.PDF]

Supplementary Materials

2. Materials and Methods

2.4. Isometric Block Tracking Motor Task

During the task, which involved visually tracking a square wave moving across a screen, a ball was visible on the monitor that reflected the amount of force the participant applied to the dynamometer. Applying grip force to the dynamometer caused the ball to move straight up on the screen, while reducing the applied force lowered it. Prior to the start of data collection, the participants were asked to squeeze the dynamometer at their maximum force, and the amount of force needed to move the ball to the top of the square wave was then calibrated to 30% of their maximum volitional contraction. The base of the square wave was calibrated to full relaxation. For most subjects, the frequency of the square wave resulted in 5 seconds of active contraction followed by 5 seconds of relaxation that repeated seven times. However, for PD08 and PD16 the square wave presented 10 seconds of active contraction followed by 10 seconds of relaxation that repeated three times. The task was switched between the hand ipsilateral and contralateral to stimulation in a pair of trials to avoid fatigue effects and evaluate bilateral physiological effects of unilateral stimulation, and a period of rest was included between each pair of task trials.

2.5. Signal Processing

EEG electrodes placed on the scalp were used to record the electrical artifact associated with delivery of each DBS pulse delivery. Using a custom MATLAB script, a 1 kHz high pass filter was applied to isolate the electrical artifact, peaks were detected, and the result was manually reviewed to ensure the selected peaks were separated by the stimulation rate (6 Hz, or about 167 milliseconds) and reflected only the electrical artifact and not spurious muscle activity or other noise.

ERPs were generated within each condition of task described above by averaging together the epochs obtained within those specific conditions. The time ranges during which each stimulation amplitude was applied were known, which allowed epochs taken at each amplitude to be averaged separately. A custom, semi-automated MATLAB script was used to segment the EMG recordings into separate behavioral states, which allowed for epochs from each state to be averaged separately. This code enveloped the rectified version of the EMG recordings of bilateral distal muscles (FCR/EDC), overlaid the aligned motor force trace from the dynamometer, then found contiguous regions exceeding a user-specified threshold to estimate periods of active muscle contraction versus relaxation. To correct any false classification of active versus inactive periods the results were manually reviewed.

3. Results

3.3. Distribution of MEPs across Proximal and Distal Upper-Extremity Musculature

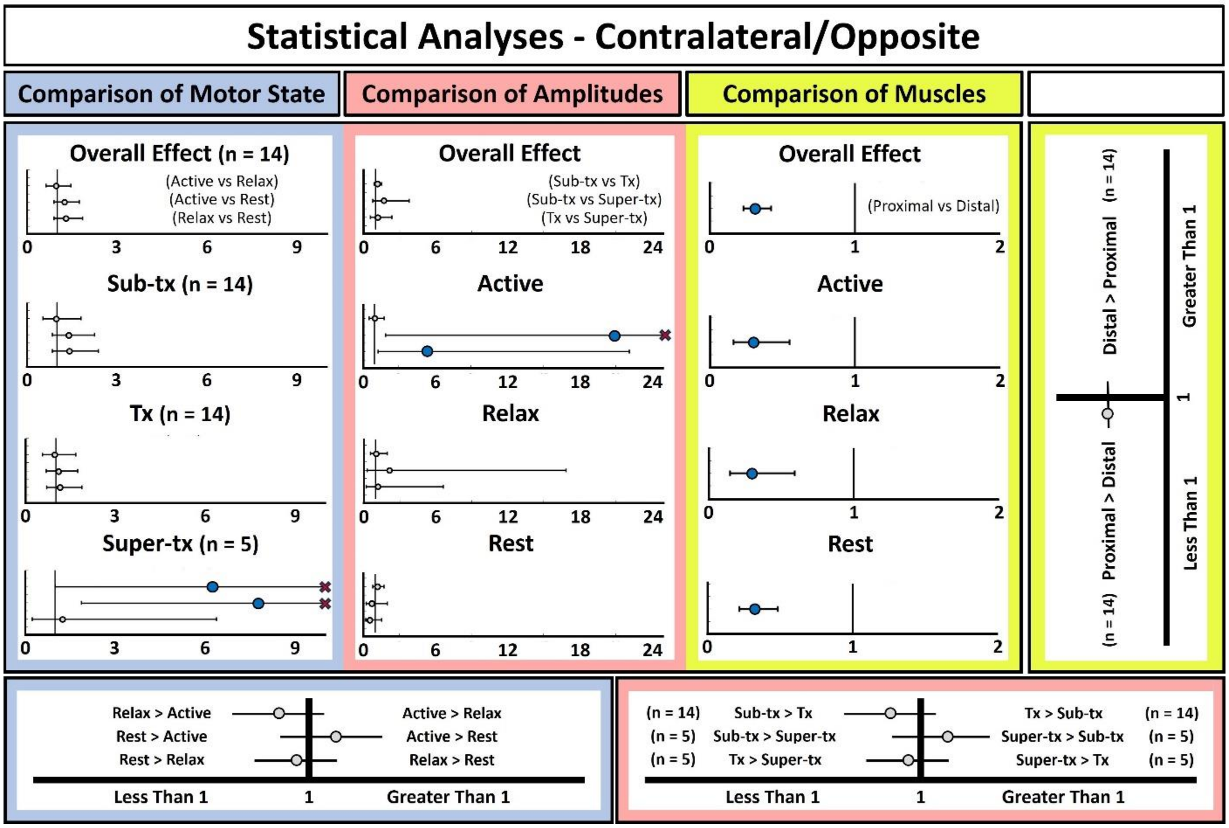

Figure S1. Effect of Activity State, Stimulation Amplitude, and Somatotopy (i.e., Muscle) on MEP Prevalence in Contralateral/Opposite condition. The overall layout of the figure is identical to **Figure 2** and is explained by the **Figure 2** caption. Large blue circles represent significant comparisons at α = 0.05, whereas small white circles are non-significant, and horizontal bands represent 95% confidence intervals. Note that all statistical comparisons involving data from the Super-tx condition have less statistical power, as there were only five subjects with such data. As such, the confidence intervals were wide and did not always fit inside the plotted range (marked by thin red X's). To see the full confidence intervals, please refer to **Table S2**.

Overall, there appears to be increases in Active > Relax & Rest in the Super-tx condition only, which corresponds to there being sensitivity to stimulation amplitude in the Active state. However, as these significant comparisons are all in the smaller (n = 5) cohort, and all comparisons from the larger (n = 14) cohort are non-significant, there is some concern that these significant comparisons may be spurious. They might be an effect of the fact that some subjects may have erroneously performed the task with both UEs simultaneously. Because of the small sample size (n = 5), it is possible that several of these subjects may have performed the task with their contralateral and ipsilateral extremities instead of just their ipsilateral extremity, meaning that the same physiology underlying the significant "Contralateral/Same" results could be erroneously biasing these comparisons. Note that this would not inappropriately bias the results in **Figure 2**, where the "Contralateral/Same" condition is being analyzed using data drawn from different time periods. However, here the conditions permitting statistical analysis with the larger sample size (n = 14) show no difference in MEP facilitation related to any condition, suggesting that a higher

sample size overcame any such bias. This includes the analysis of proximal versus distal muscles (conducted at n = 14), where there is consistently more MEP facilitation in proximal muscles than in distal muscles (i.e., similar effect size) regardless of motor state unlike the findings in **Figure 2**.

Table S1. Full "Overall Effect" Results Comparing Activity States

Overall Effect	Contralateral/Same				Contralateral/Opposite			
Subjects with Sub-tx/Tx (n = 14) *	Effect Size	95% CI Lower Bound	95% CI Upper Bound	P-value versus α = .05	Effect Size	95% CI Lower Bound	95% CI Upper Bound	P-value versus α = .05
Active versus Relax	2.106	1.386	3.199	**0.0005**	0.964	0.638	1.455	0.861
Active versus Rest	1.88	1.365	2.589	**0.0001**	1.244	0.891	1.737	0.2
Relax versus Rest	0.893	0.613	1.301	0.555	1.291	0.901	1.85	0.164
Subjects with Super-tx Only (n = 5)	Effect Size	95% CI Lower Bound	95% CI Upper Bound	P-value versus α = .05	Effect Size	95% CI Lower Bound	95% CI Upper Bound	P-value versus α = .05
Active versus Relax	2.816	1.226	6.464	**0.015**	1.516	0.59	3.895	0.387
Active versus Rest	2.923	1.547	5.523	**0.001**	1.2	0.581	2.478	0.621
Relax versus Rest	1.038	0.481	2.241	0.924	0.792	0.347	1.807	0.578

Statistically significant p-values are in bold text and are highlighted green.

* Note that the analysis of the cohort of subjects with sub-tx and tx (n = 14) excludes any data obtained at the super-tx amplitude in determining the "overall effect" but includes a larger number of subjects. However, the results obtained for the smaller cohort of subjects with super-tx data (n = 5) includes data from all three stimulation amplitudes in determining the "overall effect". The statistical interpretation of the results obtained for both cohorts is identical.

Table S2. Full Displayed Statistical Results

Comparison of Activity States	Contralateral/Same				Contralateral/Opposite			
Overall Effect	Odds Ratio	95% CI Lower Bound	95% CI Upper Bound	P-value versus α = .05	Odds Ratio	95% CI Lower Bound	95% CI Upper Bound	P-value versus α = .05
Active vs Relax (n = 14)	2.106	1.386	3.199	**0.0005**	0.964	0.638	1.455	0.861
Active vs Rest (n = 14)	1.88	1.365	2.589	**0.0001**	1.244	0.891	1.737	0.2
Relax vs Rest (n = 14)	0.893	0.613	1.301	0.555	1.291	0.901	1.85	0.164
At Sub-Tx Amplitude								
Active vs Relax (n = 14)	1.653	0.89	3.069	0.112	0.979	0.534	1.794	0.944
Active vs Rest (n = 14)	1.459	0.915	2.326	0.113	1.387	0.854	2.252	0.186
Relax vs Rest (n = 14)	0.883	0.514	1.516	0.651	1.417	0.843	2.383	0.188
At Tx Amplitude								
Active vs Relax (n = 14)	2.501	1.42	4.404	**0.002**	0.954	0.547	1.665	0.868
Active vs Rest (n = 14)	2.249	1.443	3.505	**0.0004**	1.083	0.684	1.715	0.734
Relax vs Rest (n = 14)	0.899	0.532	1.519	0.69	1.135	0.69	1.868	0.618
At Super-Tx Amplitude								
Active vs Relax (n = 5)	5.957	1.148	30.903	**0.034**	6.235	1.006	38.629	**0.049**
Active vs Rest (n = 5)	7.445	2.058	26.929	**0.003**	7.759	1.87	32.192	**0.005**
Relax vs Rest (n = 5)	1.25	0.278	5.621	0.769	1.244	0.243	6.37	0.791
Comparison of Stimulation Amplitudes	Contralateral/Same				Contralateral/Opposite			
Overall Effect	Odds Ratio	95% CI Lower Bound	95% CI Upper Bound	P-value versus α = .05	Odds Ratio	95% CI Lower Bound	95% CI Upper Bound	P-value versus α = .05
Tx vs Sub-tx (n = 14)	1.349	1.025	1.776	**0.033**	1.142	0.864	1.508	0.35
Super-tx vs Sub-tx (n = 5)	1.385	0.648	2.96	0.4	1.686	0.749	3.796	0.207
Super-tx vs Tx (n = 5)	0.692	0.362	1.32	0.263	1.163	0.571	2.37	0.676
In Active State								
Tx vs Sub-tx (n = 14)	2.017	1.126	3.614	**0.019**	1.024	0.583	1.801	0.933
Super-tx vs Sub-tx (n = 5)	3.935	0.878	17.632	0.073	20.913	1.917	228.174	**0.013**
Super-tx vs Tx (n = 5)	1.543	0.478	4.986	0.465	5.365	1.3	22.145	**0.021**
In Relax State								
Tx vs Sub-tx (n = 14)	1.127	0.605	2.1	0.705	1.034	0.547	1.953	0.919
Super-tx vs Sub-tx (n = 5)	4.292	0.395	46.573	0.228	2.168	0.279	16.859	0.455
Super-tx vs Tx (n = 5)	0.645	0.15	2.777	0.551	1.215	0.223	6.621	0.82
In Rest State								
Tx vs Sub-tx (n = 14)	1.181	0.814	1.713	0.379	1.181	0.814	1.713	0.379
Super-tx vs Sub-tx (n = 5)	0.711	0.253	2.002	0.517	0.711	0.253	2.002	0.517
Super-tx vs Tx (n = 5)	0.565	0.21	1.519	0.256	0.565	0.21	1.519	0.256

Significant p-values <0.05: bold, highlighted green. Non-significant p-values <0.10: highlighted yellow.

Upper limits of 95% confidence intervals which exceed the plotted range in **Figure 2** are highlighted red.

The designation "n = 5" or "n = 14" refers to which cohort of subjects the comparison was made from, where "n = 5" is only subjects who have super-tx data and "n = 14" is all subjects with sub-tx/tx data.

3.5. Temporal Distribution and Onset/Offset Latencies of MEP Waveforms across Muscles

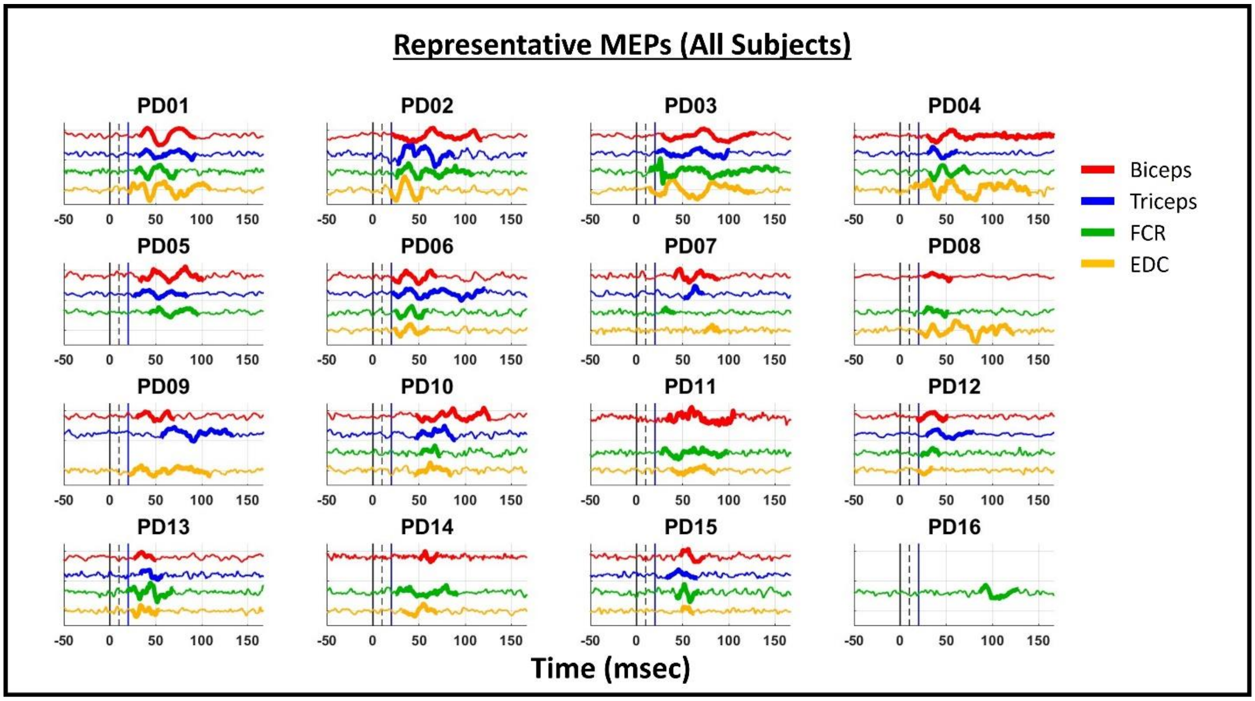

Figure S2. Representative CUE MEP Responses in All Participants. One representative MEP response is plotted for each muscle in every participant. The biceps muscle is shown in red (top waveform), the triceps muscle is shown in blue (upper-middle waveform), the FCR muscle is shown in green (lower-middle waveform), and the EDC muscle is shown in orange (bottom waveform). MEP responses were drawn from all stimulation amplitudes and motor states (i.e., not necessarily from the therapeutic amplitude condition only); the amplitude/motor state for each response is not shown. This, for example, explains why PD16 has a response in FCR despite having zero MEP responses at therapeutic amplitude, as this lone response was obtained using a different amplitude. For PD06, all responses were obtained from the follow-up recording session. For PD15, the biceps, triceps, and EDC responses were obtained from the initial recording session (i.e., during right hemispheric STN DBS) and the FCR response was obtained from the follow up session (i.e., during left hemispheric STN DBS).

This figure allows visualization of MEP responses in the participants with lower MEP prevalence values. For some participants, the MEPs have later onset times (30-50 msec), and oftentimes, the MEPs also do not last as long, with earlier offset times (50-100 msec) as well. However, even for MEPs with delayed onset times (e.g., PD09 Triceps, PD10 all muscles, PD11 EDC, and PD15 all muscles), the morphology of these MEPs appears to be concordant with MEPs in other muscles, with elicitation occurring in an overlapping time-period and with similar component (i.e., peak/trough) latencies.

3.7. Other Factors Which May Influence MEP Formation

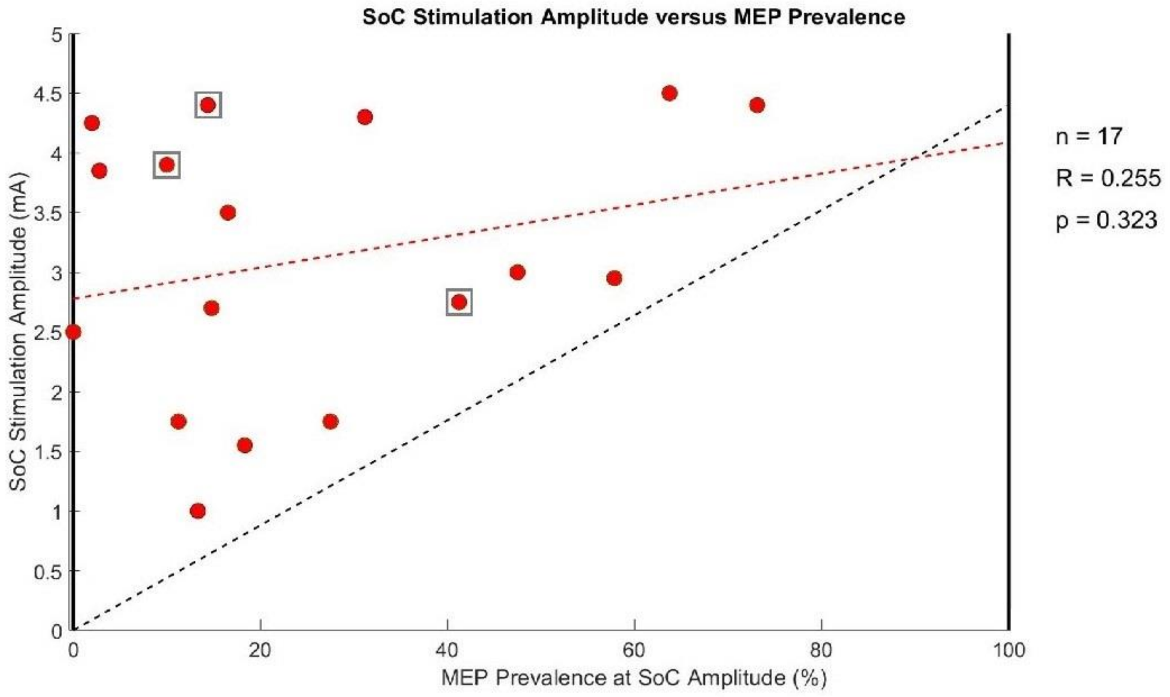

Figure S3. Therapeutic (SoC) Stimulation Amplitude versus MEP Prevalence observed at Therapeutic Amplitude. Each subject's therapeutic stimulation amplitude is plotted against the prevalence of observed MEPs at that same amplitude, which shows that all subjects with a higher MEP prevalence all had relatively higher therapeutic amplitudes (**left subplot**). In fact, the black, dotted line shows that below a certain therapeutic amplitude, there were no subjects with MEP prevalence higher than a certain value. The red, dotted line is the linear regression best-fit line. The thin black boxes represent subjects with directional contact configurations, where current steering could have played a role (**Figure S4**).

Regarding the correlation between therapeutic (SoC) stimulation amplitude and therapeutic-amplitude DBS-MEP prevalence, although the resulting Spearman's correlation is non-significant, there is certainly a significant pattern occurring in the sense that there is a "lower bound" on the therapeutic amplitude for any given observed MEP prevalence (black dotted line). In other words, no participant with >30% MEP prevalence had a therapeutic amplitude of < 2.75 mA. Recent work has shown that MEP formation increases during bipolar DBS above 3 mA (Campbell et al., 2023, Testini et al., 2025). However, at the same time, there is not an "upper bound" on the therapeutic amplitude for any given observed MEP prevalence, as some subjects with high clinical amplitudes still had low MEP prevalence. Although the cause of this is unclear, it may be associated with, for example, lead placement far from IC in these patients.

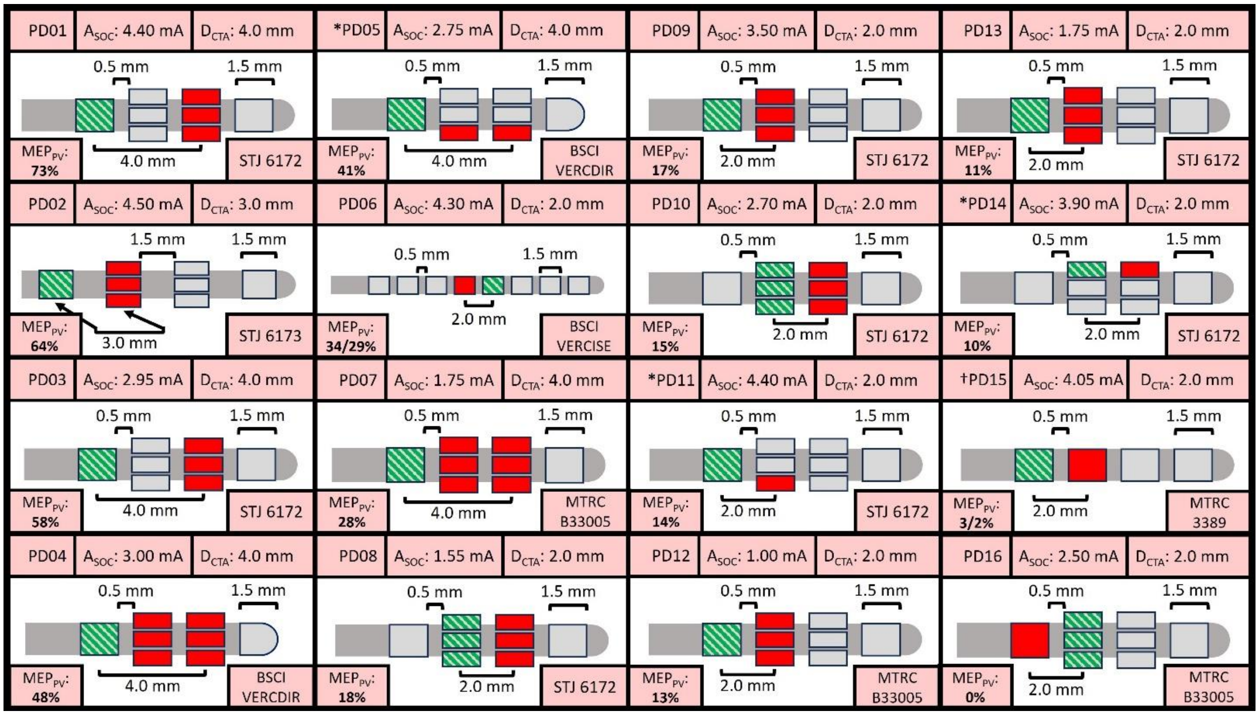

Figure S4. Subject Lead Specifications and Contact Configuration versus Results. Pictured above is the DBS lead type (manufacturer and model) for each subject, alongside the bipolar-converted standard-of-care contact configuration (cathode is red; anode is green, striped). The subjects are arranged in order, such that subjects with higher MEP prevalence are pictured on the left. Also shown is the standard of care stimulation amplitude (A_{SOC}), the MEP prevalence during DBS at SoC Amp (MEP_{PV}), and the distance between cathode and anode (D_{CTA}), which is defined as the distance between the center of the anode and the center of the farthest row of the cathode along the axis of the electrode. D_{CTA} is also denoted in the electrode illustration, alongside the contact width and inter-contact longitudinal space width. The manufacturer and model of each DBS lead is also expressly denoted in written form, with the following abbreviations: STJ = St. Jude; MTRC = Medtronic; BSCI = Boston Scientific; VERCDIR = Vercise Directed (a.k.a., Cartesia); VERCISE = Vercise Non-directional. The three subjects with directional contact configurations are denoted by an asterisk; for these three subjects, current steering could have played a role in influencing MEP_{PV}.

†Note that PD15, despite being stimulated in different hemispheres at slightly different amplitudes during the initial and follow up sessions, had the same lead model and same contact configuration during both sessions. As such, the two sessions for PD15 are not shown separately above, and the A_{SOC} is averaged.

The most salient takeaway from this figure is that the more responsive subjects (i.e., higher MEP_{PV}) had larger D_{CTA} values. Six of the top seven responders had a D_{CTA} >= 3 mm, whereas the remaining subjects had a D_{CTA} of 2 mm (i.e., adjacent contact rows for cathode/anode). Changes in field geometry based on stimulation with more ventral contacts induce stronger MEPs (Ashby et al., 1999, Testini et al., 2025).

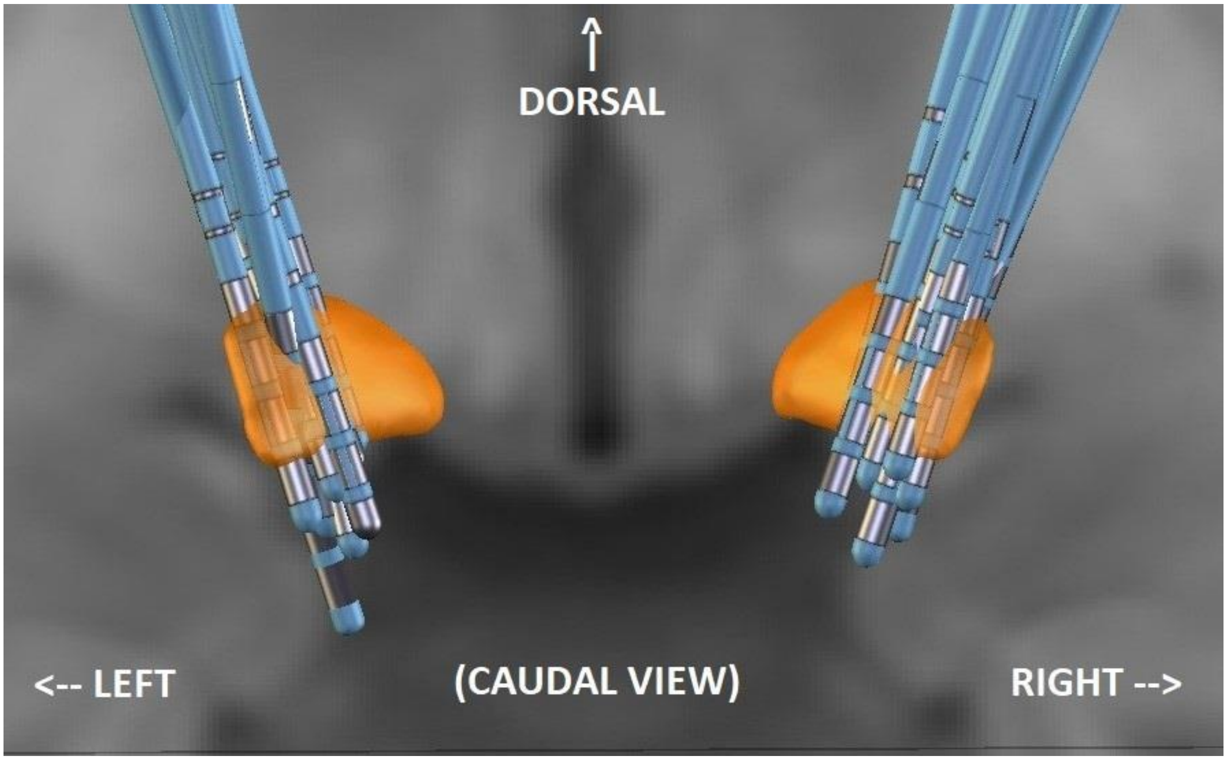

Figure S5. Estimated Lead Location Relative to the STN (orange) for Study Participants. 3D images were rendered in MNI 152 Nonlinear 2009b Asymmetric space as reconstructed in Lead-DBS. The rendered STN volume is based on the DISTAL atlas (Ewert et al., 2018), with the figure depicting a caudal view (i.e., as viewed from a posterior orientation).

Although inter-subject variability was present, the image supports clinical targeting of the dorsolateral STN across all participants. Stimulation of this area of STN has been shown to be optimal for maximizing clinical improvement (Horn et. al., 2017). Moreover, the estimates support that the electrophysiology data presented were derived from stimulation delivered in the area of this dorsolateral, sensorimotor subregion of the STN.

References:

1. Ashby P, Kim YJ, Kumar R, Lang AE, Lozano AM. Neurophysiological effects of stimulation through electrodes in the human subthalamic nucleus. Brain. 1999 Oct;122 (Pt 10):1919-31. doi: 10.1093/brain/122.10.1919. PMID: 10506093.
2. Campbell BA, Favi Bocca L, Tiefenbach J, Hogue O, Nagel SJ, Rammo R, Escobar Sanabria D, Machado AG, Baker KB. Myogenic and cortical evoked potentials vary as a function of stimulus pulse geometry delivered in the subthalamic nucleus of Parkinson's disease patients. Front Neurol. 2023 Aug 24;14:1216916. doi: 10.3389/fneur.2023.1216916. PMID: 37693765; PMCID: PMC10484227.
3. Chen R, Yung D, Li JY. Organization of ipsilateral excitatory and inhibitory pathways in the human motor cortex. J Neurophysiol. 2003 Mar;89(3):1256-64. doi: 10.1152/jn.00950.2002. Epub 2002 Oct 30. PMID: 12611955.
4. Ewert S, Plettig P, Li N, Chakravarty MM, Collins DL, Herrington TM, Kühn AA, Horn A. Toward defining deep brain stimulation targets in MNI space: A subcortical atlas based on multimodal MRI, histology and structural connectivity. Neuroimage. 2018 Apr 15;170:271-282. doi: 10.1016/j.neuroimage.2017.05.015. Epub 2017 May 20. PMID: 28536045.
5. Horn A, Neumann WJ, Degen K, Schneider GH, Kühn AA. Toward an electrophysiological "sweet spot" for deep brain stimulation in the subthalamic nucleus. Hum Brain Mapp. 2017 Jul;38(7):3377-3390. doi: 10.1002/hbm.23594. Epub 2017 Apr 8. PMID: 28390148; PMCID: PMC6867148.
6. Taga M, Charalambous CC, Raju S, Lin J, Zhang Y, Stern E, Schambra HM. Corticoreticulospinal tract neurophysiology in an arm and hand muscle in healthy and stroke subjects. J Physiol. 2021 Aug;599(16):3955-3971. doi: 10.1113/JP281681. PMID: 34229359; PMCID: PMC8942144.
7. Testini P, Wang A, Cole E, Miocinovic S. Motor evoked potentials as a side effect biomarker for deep brain stimulation programming. medRxiv [Preprint]. 2025 Jan 27:2025.01.24.25320924. doi: 10.1101/2025.01.24.25320924. PMID: 39974135; PMCID: PMC11838958.
8. Ziemann U, Ishii K, Borgheresi A, Yaseen Z, Battaglia F, Hallett M, Cincotta M, Wassermann EM. Dissociation of the pathways mediating ipsilateral and contralateral motor-evoked potentials in human hand and arm muscles. J Physiol. 1999 Aug 1;518 (Pt 3)(Pt 3):895-906. doi: 10.1111/j.1469-7793.1999.0895p.x. PMID: 10420023; PMCID: PMC2269467.
